# Regional Analysis of the Structural Availability of Physical and Rehabilitation Medicine Services Funded by the National Health Insurance Fund for Patients with Rare Diseases in Bulgaria

**DOI:** 10.3390/healthcare14121691

**Published:** 2026-06-12

**Authors:** Evelina Razheva, Georgi Iskrov, Tsonka Miteva-Katrandzhieva, Rumen Stefanov

**Affiliations:** 1 Department of Social Medicine and Public Health, Faculty of Public Health, Medical University-Plovdiv, 4000 Plovdiv, Bulgaria; georgi.iskrov@mu-plovdiv.bg (G.I.); tsonka.miteva@mu-plovdiv.bg (T.M.-K.); rumen.stefanov@mu-plovdiv.bg (R.S.); 2 Institute for Rare Diseases, 4017 Plovdiv, Bulgaria

**Keywords:** rare diseases, rehabilitation, physical and rehabilitation medicine, health services availability, regional disparities, Bulgaria

## Abstract

Background: Rare diseases are associated with chronic progression, functional impairment, and complex care needs, requiring long-term and coordinated rehabilitation. Physical and Rehabilitation Medicine (PRM) plays a key role in maintaining functional capacity and improving quality of life; however, access to rehabilitation services remains uneven across regions. Aim: This study aims to assess the regional structural availability of PRM services across Bulgaria and to identify territorial differences in the organizational profile of rehabilitation services that may influence the potential availability of rehabilitation care for patients with rare diseases. Methods: A descriptive cross-sectional study was conducted using publicly available aggregated data from the NHIF and the National Statistical Institute as of 31 December 2024. Structural indicators included the number of outpatient and inpatient PRM healthcare facilities and PRM specialists, standardized per 100,000 population, as well as the outpatient-to-inpatient facility ratio (OFs/IFs). Hierarchical cluster analysis (Ward’s method, Euclidean distance) was applied as an exploratory tool to identify similarities in regional service availability profiles. Results: Substantial regional differences in the structural availability of PRM services were identified. Outpatient facilities ranged from 4.46 to 6.74 per 100,000 population, while inpatient facilities ranged from 2.30 to 3.42 per 100,000 population. The OFs/IFs ratio varied between 1.30 and 2.26, indicating different organizational profiles of PRM service provision. Exploratory hierarchical clustering suggested two broad regional service profiles: one characterized by a relatively balanced distribution of outpatient and inpatient structures and another characterized by a predominance of outpatient-oriented rehabilitation services. Conclusion: The findings reveal substantial regional differences in the organization of PRM services in Bulgaria. Regions with a predominance of outpatient structures may demonstrate different capacities for delivering comprehensive rehabilitation services, particularly for patients with complex long-term needs, including rare diseases. The results highlight the need for targeted regional planning, improved integration of rehabilitation services, and policy measures aimed at ensuring equitable access to care.

## 1. Introduction

Rare diseases represent a significant public health priority due to their low prevalence, chronic course, and high risk of long-term disability and premature mortality. Global analyses indicate that rare diseases affect more than 300 million people worldwide and are associated with complex medical and social needs that are difficult to address within standard healthcare systems. A lack of expertise, fragmented services, and territorial inequalities contribute to delayed diagnosis, limited access to treatment, and poorer functional outcomes [1,2,3].

Physical and Rehabilitation Medicine (PRM) is a key component of tertiary prevention in rare diseases, aiming to maintain functional capacity, reduce disability, and support social and occupational integration. International studies highlight that many individuals with rare diseases face barriers to accessing rehabilitation services, including limited availability of specialists, geographic remoteness, and lack of coordinated care. These challenges are often compounded by financial barriers, such as high out-of-pocket costs, limited resources for long-term rehabilitation, and accumulation of indirect costs for families, particularly in resource-constrained regions and among patients with severe functional impairment [4,5,6,7,8,9].

In recent years, growing evidence has documented regional disparities in the availability and utilization of rehabilitation services across different countries, including those with well-developed healthcare systems. Numerous studies report substantial interregional variation in the number of rehabilitation providers and the volume of services delivered, raising concerns about the need for more equitable resource planning and allocation. Such territorial inequalities are often associated with differences in demographic structure, degree of urbanization, concentration of hospital and academic centers, and the level of integration of rehabilitation within healthcare systems [10,11,12,13].

In Bulgaria, rehabilitation services are primarily funded by the National Health Insurance Fund (NHIF) and delivered through both inpatient and outpatient structures. However, the absence of a dedicated and clearly defined rehabilitation framework for patients with rare diseases complicates service planning and may contribute to regional differences in the availability of rehabilitation services, particularly outside major urban centers and specialized facilities. Existing national policy documents and scientific evidence suggest that patients frequently encounter difficulties in accessing comprehensive and long-term rehabilitation. Despite this, there is a lack of systematic analyses of the regional availability of PRM structures funded by the NHIF in the context of rare diseases and the broader agenda for strengthening rehabilitation within healthcare systems [14,15].

Healthcare accessibility may also be assessed through healthcare catchment area approaches, which define the geographic areas from which patients are likely to seek services and incorporate travel distance, travel time, and mobility patterns into accessibility assessment. Such approaches may provide a more realistic representation of healthcare accessibility than analyses based solely on administrative boundaries. However, their application requires detailed patient-level utilization and mobility data that are not currently available for rehabilitation services in Bulgaria [16].

## 2. Materials and Methods

### 2.1. Study Design and Data Sources

A descriptive cross-sectional study was conducted to assess the availability of PRM services funded by the NHIF across the economic regions of Bulgaria as of 31 December 2024. Structural indicators related to the availability and distribution of healthcare facilities and PRM specialists were analyzed as proxies for potential access to rehabilitation services for patients with rare diseases.

Publicly available aggregated data were obtained from:

- The National Health Insurance Fund (registers of outpatient and inpatient healthcare providers contracted in PRM) [17,18];
- The National Statistical Institute (NSI), including demographic data and classification of economic regions in Bulgaria [19,20].

The analysis covers the six economic regions of the Republic of Bulgaria: Northwestern (Vidin, Vratsa, Lovech, Montana, Pleven), North Central (Veliko Tarnovo, Gabrovo, Razgrad, Ruse, Silistra), Northeastern (Varna, Dobrich, Targovishte, Shumen), Southeastern (Burgas, Sliven, Stara Zagora, Yambol), Southwestern (Blagoevgrad, Kyustendil, Pernik, Sofia Province, Sofia City) and South Central (Kardzhali, Pazardzhik, Plovdiv, Smolyan, Haskovo).

The following indicators were included in the analysis:

- Number of PRM specialists by region;
- Number of outpatient healthcare facilities (OFs) in PRM contracted with NHIF;
- Number of inpatient healthcare facilities (IFs) providing PRM services;
- Ratio of OFs to IFs as an indicator of the structural profile of rehabilitation services.

Where applicable, indicators were standardized per 100,000 population to ensure comparability across regions.

Due to the lack of nationally available data specifically describing rehabilitation service utilization among patients with rare diseases, structural indicators were used as proxies for potential access to PRM services for this population.

### 2.2. Statistical Methods

Descriptive statistics were used to summarize the main characteristics of the indicators across regions (absolute values, rates per 100,000 population, and ratios). Hierarchical cluster analysis (Ward’s method, Euclidean distance) was applied as an exploratory method to identify regions with similar structural profiles.

The cluster analysis applied in the present study was intended as an exploratory approach to identify similarities in the structural profile of PRM service provision across the six economic regions of Bulgaria. Although spatially explicit analytical approaches may provide additional insight into territorial interactions and geographic continuity, the present analysis focused primarily on structural healthcare indicators and regional organizational patterns. The objective was not to identify spatial clusters, geographically contiguous territories, or patterns of patient mobility, but rather to summarize similarities in regional service availability profiles based on the selected indicators. Given the limited number of territorial units and the exploratory scope of the study, conventional hierarchical clustering was considered appropriate for an initial assessment of regional differences.

Due to the limited number of territorial units included in the analysis, inferential statistical procedures were not applied, and findings were interpreted descriptively.

Data processing was performed using Microsoft Excel, and statistical analyses were conducted in IBM SPSS Statistics v23.00.

Ethical approval was not required for this study, as it was based entirely on aggregated data from publicly accessible sources and official documents. The research did not include the collection, processing, or analysis of individual-level patient data, nor did it involve any personally identifiable or sensitive health information. Consequently, review or approval by a Research Ethics Committee was not necessary.

## 3. Results

### 3.1. Descriptive Regional Data

Standardized indicators reveal substantial regional disparities in the availability of PRM healthcare facilities across the six regions of Bulgaria (Table 1).

**Figure 1:**
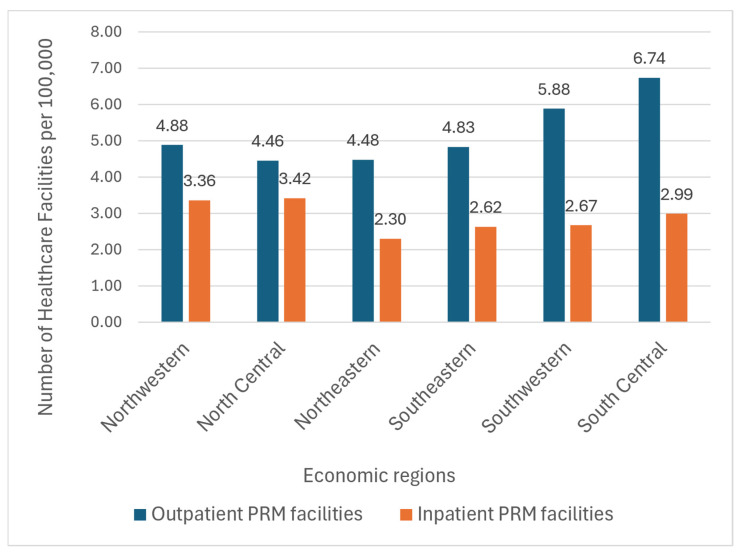
PRM Healthcare Facilities per 100,000 population by region.

The number of outpatient PRM facilities per 100,000 population ranges from 4.46 in the North Central region to 6.74 in the South Central region, while inpatient facilities vary from 2.30 in the Northeastern region to 3.42 in the North Central region (Figure 1).

**Figure 2:**
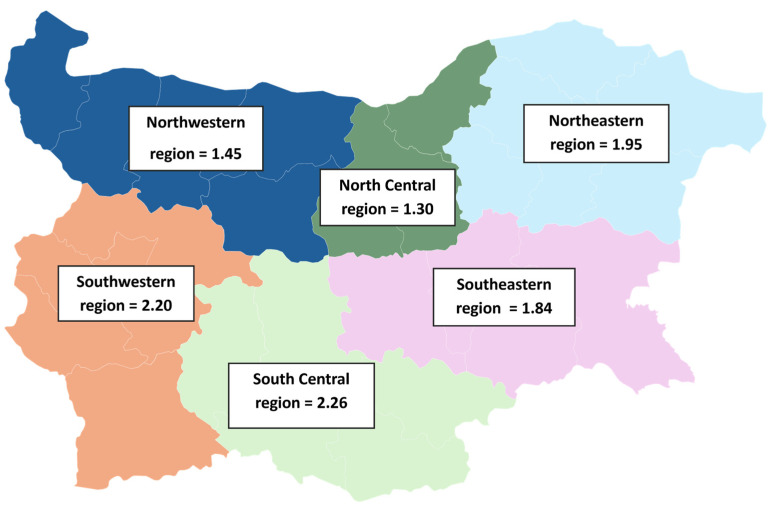
Outpatient-to-inpatient PRM facility ratio (OFs/IFs) across the economic regions of Bulgaria, 2024.

The ratio between outpatient and inpatient PRM structures (OFs/IFs) ranges from 1.30 in the North Central region to 2.26 in the South Central region, indicating different structural profiles of rehabilitation services across regions. As shown in Figure 2, the OFs/IFs ratio varies substantially across regions, with higher values observed in the South Central and Southwestern regions. Values correspond to the OFs/IFs ratio for each economic region, as labeled on the map.

**Figure 3:**
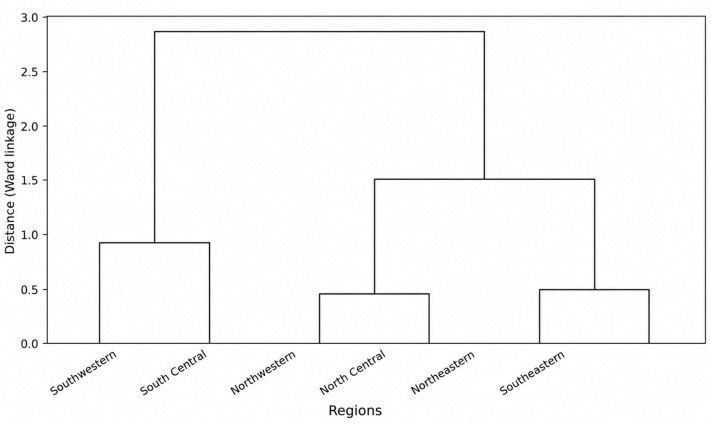
Hierarchical clustering dendrogram of Bulgarian regions. Dendrogram illustrating the exploratory regional grouping of the six economic regions of Bulgaria based on PRM service availability indicators.

As illustrated in Figure 3, the exploratory hierarchical clustering procedure identified two broad regional service profiles based on the structural characteristics of PRM service provision. The first regional profile includes the Northwestern and North Central regions, characterized by a relatively balanced distribution of outpatient and inpatient PRM services. The second regional profile comprises the Northeastern, Southeastern, Southwestern, and South Central regions, where outpatient services predominate and indicators show similar structural patterns.

The interpretation of two regional profiles was based on the visual structure of the dendrogram and the relative distance between regional groupings identified through Ward’s linkage method. The two-profile solution was considered the most interpretable representation of the observed structural differences in PRM service organization across the economic regions.

### 3.2. Differences Between Regional Service Profiles

The exploratory regional grouping revealed different structural profiles of PRM service organization. The Northwestern and North Central regions demonstrated a relatively balanced distribution between outpatient and inpatient PRM facilities. In contrast, the Northeastern, Southeastern, Southwestern, and South Central regions showed a predominance of outpatient-oriented rehabilitation structures and higher OFs/IFs ratios. The grouping was used as a descriptive tool to summarize similarities among regions and support the interpretation of regional service profiles rather than to establish statistically robust territorial classifications. Due to the limited number of territorial units included in the analysis, these findings should be interpreted as exploratory and descriptive. These profiles should not be interpreted as spatial clusters but rather as descriptive regional groupings based on similarities in service availability indicators.

## 4. Discussion

The present study suggests pronounced territorial inequalities in the availability and structural organization of PRM services in Bulgaria. These findings reflect broader systemic challenges in the distribution of healthcare resources and are consistent with international evidence highlighting persistent disparities in rehabilitation service provision across Europe [21,22].

The observed differences may also be explained by the demographic profile, level of urbanization, and historical development of the hospital network in Bulgaria. Regions with larger urban centers and more concentrated hospital infrastructure are more likely to provide access to comprehensive inpatient rehabilitation programs, whereas in predominantly rural or peripheral areas, the development of outpatient PRM practices may be more feasible and economically sustainable. Similar associations between territorial inequalities, demographic challenges, and the concentration of healthcare resources have been described in European regional analyses, where insufficient investment and fragmented policies further exacerbate disparities in access to services and social support [8,22,23].

International literature shows that people with rare diseases are often disproportionately affected by geographic barriers and a lack of coordinated care. European studies on patient pathways for rare diseases emphasize that many patients must travel to a limited number of expert centers, increasing inequalities for individuals living in peripheral or resource-limited regions. Moreover, analyses of access to diagnosis and treatment for rare diseases indicate that organizational and geographic barriers frequently coincide with a lack of integrated services, including rehabilitation, resulting in incomplete addressing of patients’ complex needs [1,2,5,6,24,25].

In this context, the pattern observed in Bulgaria—where four of the six economic regions are characterized by a predominance of outpatient-oriented PRM structures—may exacerbate the challenges faced by patients with rare diseases who require highly specialized and multidisciplinary rehabilitation. Global and European reviews emphasize that the lack of integrated rehabilitation programs remains a key gap in comprehensive care, particularly in countries and regions with limited resources [1,3,14]. This suggests that the regional variation in the structural organization of PRM services may indicate differences in the regional capacity to provide comprehensive rehabilitation services, affecting not only geographic accessibility but also the quality and continuity of care provided to this patient group.

The Southwestern and South Central regions exhibited distinctive structural characteristics, including larger population concentrations and higher outpatient-to-inpatient facility ratios compared with the remaining regions. However, this pattern was not fully reflected in the distribution of PRM specialists per 100,000 population, as the Southwestern region did not demonstrate the same relative advantage for workforce availability. This discrepancy suggests that the territorial distribution of healthcare facilities and the distribution of specialized rehabilitation professionals do not necessarily follow identical regional patterns and should therefore be interpreted separately when assessing regional service capacity. Therefore, facility availability and workforce availability should be considered complementary but distinct dimensions of regional rehabilitation capacity.

At the same time, it should be emphasized that the present analysis is based on structural indicators used as proxies for potential access to services. Although this approach does not capture actual service utilization, rehabilitation intensity, or clinical outcomes, it provides important insight into system capacity and regional readiness to deliver PRM services. Therefore, the findings should be interpreted as an assessment of healthcare system capacity rather than direct evidence of individual access. Furthermore, the present study did not include spatial accessibility analyses, healthcare catchment area modeling, or patient mobility assessment. Healthcare catchment areas represent the geographic areas from which patients are likely to seek healthcare services and provide an alternative framework for evaluating accessibility beyond administrative boundaries. Recent studies have proposed methods combining spatial and temporal distance measures to define healthcare catchment areas and examine patient mobility patterns, thereby providing a more realistic representation of service accessibility and healthcare utilization. However, the application of such approaches requires detailed patient-level utilization and mobility data that were not available for the present study. Future research incorporating GIS-based methods, catchment area analyses, and rehabilitation utilization data could provide a more comprehensive understanding of accessibility patterns among patients with rare diseases [16].

For patients with rare diseases, a balanced model of PRM service provision (as observed in Regional Profile 1) is more closely aligned with the recommendations of European Reference Networks and national rare disease strategies, which emphasize coordination between expert centers, inpatient rehabilitation, and local outpatient services. In Regional Profile 2, where outpatient structures predominate, further research is needed to determine whether existing capacity is sufficient to manage complex cases and to what extent patients have real access to inpatient PRM without significant financial or logistical barriers, including the need to travel to other regions or countries [2,24,25,26,27].

Policy recommendations formulated in international and European documents on rare diseases emphasize a network-based approach, promoting coordinated and regionally distributed structures that ensure integrated care, including rehabilitation, close to patients’ place of residence. The present findings may serve as a basis for such planning in Bulgaria by identifying regions with structural imbalances between outpatient and inpatient PRM services and guiding targeted investments in line with the principles of health equity and European rare disease policies [1,2,26,28,29].

Such an approach would allow rehabilitation planning to move beyond the simple availability of services and toward a more equitable model of integrated, needs-based care.

### Limitations

This study has several limitations. First, it relies on aggregated structural indicators and does not include data on actual service utilization, waiting times, or clinical outcomes, which limits the ability to assess real-world accessibility and effectiveness.

Second, the analysis is based on only six economic regions, which limits statistical power and generalizability. Consequently, the regional grouping should be interpreted as exploratory and descriptive rather than confirmatory. The grouping was not externally validated and may be sensitive to small variations in the underlying indicators due to the limited number of territorial units included in the analysis. Therefore, the identified regional profiles should not be interpreted as spatial clusters or territorial classifications but rather as descriptive groupings of regions with similar structural characteristics.

Third, this study does not account for privately funded rehabilitation services or provide specific data on patients with rare diseases. Therefore, the findings reflect potential availability of services rather than actual access at the individual level.

Additionally, this study did not incorporate spatial analytical techniques, geographic distance measures, healthcare catchment area modeling, or cross-regional mobility analyses. Consequently, territorial accessibility patterns beyond administrative regional boundaries could not be assessed.

Furthermore, this study remains primarily descriptive and exploratory and does not include formal analytical modeling of determinants associated with regional disparities in PRM service provision.

Despite these limitations, this study provides valuable evidence regarding regional differences in the structural availability and organization of PRM services and may serve as a starting point for future analyses incorporating spatial accessibility methods, patient-level utilization data, and more advanced analytical approaches.

## 5. Conclusions

This study provides a targeted analysis of the regional availability of PRM services funded by the National Health Insurance Fund across Bulgaria’s six economic regions, with a focus on potential accessibility for patients with rare diseases. The results reveal pronounced territorial differences in the organization of rehabilitation care and identify two broad regional service profiles—one with a more balanced distribution between outpatient and inpatient PRM services, and another characterized by a clear predominance of outpatient structures.

These structural differences may indicate variations in the regional capacity to provide comprehensive and multidisciplinary rehabilitation services for patients with complex long-term needs, including individuals with rare diseases. This is particularly critical for individuals with rare diseases, who frequently require prolonged, multidisciplinary, and coordinated rehabilitation programs—needs that are difficult to meet in regions with limited inpatient capacity or a fragmented service network. In this context, the identified regional imbalances suggest the presence of regional variation in the structural organization of rehabilitation services across Bulgaria.

From a health policy perspective, these findings underscore the need for more precise regional planning of rehabilitation services, taking into account the specific needs of vulnerable groups such as patients with rare diseases. Identifying regions with different PRM service profiles may serve as a basis for prioritizing investments, developing inpatient and multidisciplinary rehabilitation units, and improving coordination between outpatient and inpatient care. Future research should incorporate data on actual service utilization, patient pathways, and clinical outcomes to more comprehensively assess the impact of regional differences on the health and quality of life of people with rare diseases.

## Figures and Tables

**Table 1:** Descriptive data by region.

Economic Region	Population as of 31 December 2024	OFs per 100,000	IFs per 100,000	PRM Specialists per 100,000	OFs/IFs Ratio
Northwestern	655,430	4.88	3.36	10.37	1.45
North Central	673,331	4.46	3.42	10.10	1.30
Northeastern	826,742	4.48	2.30	10.40	1.95
Southeastern	952,383	4.83	2.62	11.86	1.84
Southwestern	2,022,982	5.88	2.67	9.69	2.20
South Central	1,306,492	6.74	2.99	11.71	2.26

## Data Availability

The data used in this study are publicly available from the National Health Insurance Fund (NHIF) and the National Statistical Institute (NSI) of Bulgaria. The datasets analyzed during the current study are available from the corresponding author upon reasonable request.

## Abbreviations

The following abbreviations are used in this manuscript:

PRM	Physical and Rehabilitation Medicine
NHIF	National Health Insurance Fund
NSI	National Statistical Institute
OFs	Outpatient healthcare facilities
IFs	Inpatient healthcare facilities
OFs/IFs	Ratio of outpatient to inpatient facilities
